# Sensitive electrochemical sensor for simultaneous determination of epinephrine and norepinephrine by SiO_2_NP@MWCNT-COOH nanoparticle-modified SPCEs

**DOI:** 10.55730/1300-0527.3903

**Published:** 2026-07-15

**Authors:** Merve YILMAZ ÇILÇAR, Melike BİLGİ KAMAÇ, Ayşenur YILMAZ KABACA, Şahin TOPARLI, Faruk TERZİOĞLU, Emir ATEŞ

**Affiliations:** 1 Department of Chemistry, Faculty of Science, Çankırı Karatekin University, Çankırı, Turkiye; 2 Department of Medical Laboratory Techniques, Şabanözü Vocational School, Çankırı, Turkiye

**Keywords:** Simultaneous detection, epinephrine, norepinephrine, silicon oxide nanoparticles, multiwalled carbon nanotubes, screen-printed carbon electrode

## Abstract

Epinephrine (EP) and norepinephrine (NEP), both neurotransmitters, are two important biomolecules that play critical roles in the body, from the central nervous system to metabolic processes. They are also important biomarkers for clinical diagnosis and monitoring. In this study, a novel, fast, practical, and high-performance electrochemical sensor based on a SiO_2_NP@MWCNT-COOH nanocomposite was developed for the simultaneous determination of EP and NEP. The nanocomposite was modified onto the surface of screen-printed electrodes (SPCEs) and its characterization was performed (SEM-EDX, XRD, FTIR, CV, DPV, and EIS). The individual and simultaneous determinations of EP and NEP were performed using square-wave voltammetry, yielding a wide linear range and low detection limits. The developed sensor successfully measured EP-NEP levels simultaneously in commercial human blood serum samples with high recovery rates. Analysis of selectivity and shelf-life demonstrated that the proposed sensor exhibited high selectivity and stability. While further validation with real clinical samples is required, this nanocomposite-based electrochemical sensor provides promising proof-of-concept for potential future use in rapid and reliable point-of-care applications.

## 1. Introduction

Epinephrine (EP) and norepinephrine (NEP), both catecholamines, are essential biomolecules that function as neurotransmitters and hormones. These biomolecules play critical roles in stress responses, cardiovascular regulation, metabolic processes, and central nervous system function. Abnormalities in EP and NEP levels are considered important biomarkers for the diagnosis and monitoring of many clinical conditions, such as Parkinson disease, depression, hypertension, and adrenal tumors [1–3]. EP and NEP are synthesized from the same metabolic pathway and measuring only one can often be misleading. Simultaneous measurement of both provides a holistic picture of catecholamine metabolism [4].

Traditional analytical methods for determining EP and NEP levels include high-performance liquid chromatography, capillary electrophoresis, spectroscopic methods, and chemiluminescence [5–7]. However, the disadvantages of these approaches generally include complex sample preparation, long analysis times, high cost, and a lack of portability for in situ or spot measurements [8]. Electrochemical techniques, on the other hand, offer advantages such as low cost, simple instrumentation, rapid measurement, high sensitivity, and ease of microscale application [9]. In particular, catecholamines such as EP and NEP can undergo direct reduction and oxidation due to their electroactivity. However, simultaneous determination of EP and NEP is difficult because they have very similar chemical structures, both possessing a catechol ring and an amine group. Since the oxidation peaks of EP and NEP are observed at similar potentials in electrochemical determination, simultaneous determination based on tracking the oxidation peak is not possible. However, the electrochemical reduction peaks of EP and NEP occur at different potentials, enabling simultaneous electrochemical determination [10]. Sufficient separation of the reduction peaks of these two catecholamines is nevertheless necessary, and the acidic/basic environment and electrolyte composition are also important [11]. Therefore, electrocatalytic reaction kinetics can be improved by using modified electrode surfaces (e.g., nanomaterials, metal oxide nanoparticles [NPs], conductive polymers, carbon nanotubes, and graphene-based materials), thereby providing lower detection limits and clearer peak separation [12].

Studies in the literature on the electrochemical determination of EP have used carbon nanotubes [13], multiwalled carbon nanotubes (MWCNTs) [14], MoS_2_ [15], ZnO [16], and reduced graphene oxide (RGO) [17] as electrode surfaces. Graphene@nickel molybdenum sulfide nanowire arrays [18] and CuO-carbon quantum dots [19] have been modified with nanomaterials. In these studies, cyclic voltammetry (CV) and differential pulse voltammetry (DPV) were generally used, yielding detection limits at the micromolar level. In the design of electrochemical NEP sensors, 3-aminophenylboronic acid and NiO-RGO have been used [20]. TiO_2_ [21] and metal–organic frameworks (MOFs) [22,23] have also been used, with reported detection limits at the micromolar level. Studies on NEP sensors have mainly been carried out using the DPV method. In recent years, the simultaneous determination of EP and NEP has been successfully performed, particularly using electrodes modified with cetyltrimethylammonium bromide (CTAB)-supported SnO_2_ NPs [10]. Similarly, electrodes modified with carbon nanomaterials (graphene, carbon nanotubes) and MOFs have also been used in sensor studies for this purpose [10,24–26]. There are a limited number of studies on the simultaneous electrochemical determination of EP and NEP. The present study aims to address this significant gap in the literature and develop a rapid, practical electrochemical sensor with potential clinical utility. The key novelty of this study is the first-ever use of a SiO_2_NP@MWCNT-COOH hybrid nanocomposite for the simultaneous determination of EP and NEP. The developed hybrid platform synergistically combines the high electrical conductivity and excellent electron transfer kinetics of MWCNT-COOH with the large surface area and superior adsorption capacity of SiO_2_ NPs. Silanol (Si–OH) groups on the SiO_2_ surface and carboxyl (−COOH) groups in MWCNTs form strong hydrogen bonds and π–π interactions with amine (−NH_2_) and hydroxyl (−OH) groups in EP and NEP molecules. These interactions enable preconcentration of the analytes on the electrode surface, thereby accelerating electron transfer and amplifying the signal. This synergistic effect, reported in the literature for similar SiO_2_/CNT hybrid systems, demonstrates that the combination of silica’s large surface area and the high conductivity of carbon nanotubes significantly enhances electrocatalytic activity [27–29]. For example, Arvand and Hassannezhad reported that an electrode modified with an Fe_3_O_4_@SiO_2_/MWCNT nanocomposite provided a 2.7-fold higher peak current in uric acid determination compared to an electrode modified with MWCNT alone, and they attributed this improvement to the synergistic effect of the nanocomposite [27]. Similarly, Dhaffouli et al. showed that CTAB@SiO_2_@MWCNT-APTES hybrid material increased the selectivity and sensitivity of a uric acid sensor by providing a large surface area and excellent compatibility [28]. In the present study, disposable screen-printed electrodes (SPCEs) were modified with a silicon oxide NP@carboxyl-functionalized MWCNT (SiO_2_NP@MWCNT-COOH) nanocomposite and their morphological, chemical, and electrochemical characterizations were performed based on scanning electron microscopy-energy dispersive X-ray spectroscopy (SEM-EDX), X-ray diffraction (XRD), Fourier transform infrared (FTIR) spectroscopy, CV, DPV, and electrochemical impedance spectroscopy (EIS). The effects of the MWCNT-COOH and SiO_2_NP on the electrochemical behavior of EP and NEP were investigated in detail. The prepared sensors were used to perform separate and simultaneous analyses of EP and NEP using square-wave voltammetry (SWV). Simultaneous EP-NEP analyses were also successfully performed on commercial human blood serum samples.

## 2. Experimental

All utilized materials, reagents, instruments, and electrochemical measurement parameters are available in the Supplementary Material File.

### 2.1. Preparation of SPCE/SiO_2_NP@MWCNT-COOH

SiO_2_NP (0.5 mg) and MWCNT-COOH (0.5 mg) were weighed and stirred in 50 mM phosphate buffer (PBS; 1 mL, pH 7.0) for 1 h (350 rpm, room temperature). The mixture was then stirred in a sonicator for 2 h to obtain a homogeneous SiO_2_NP@MWCNT-COOH composite. The SiO_2_NP@MWCNT-COOH composite (3 μL) was dropped onto the SPCE and dried in the dark at room temperature [30].

## 3. Result and discussion

### 3.1. SiO_2_NP@MWCNT-COOH modification on SPCEs

Modifying NPs by physical adsorption is simple and practical. However, if there are not enough NPs during modification, the expected electronic conductivity cannot be achieved, and if there are too many NPs, it causes nonfaradic current [30,31]. For this purpose, CV measurements were performed for SiO_2_NP@MWCNT-COOH-modified SPCEs with different layer numbers (1–2–3 layer-by-layer [LBL]) in redox probe solution at different scan rates (10–125 mV s^−1^). Voltammograms and Ipa-*v*^1/2^ plots are given in Supplementary Figures SA–SF. The correlation coefficients of the plotted graphs are close to 1, indicating that the electrochemical process occurring on the electrode surface in the redox probe solution was diffusion-controlled.

For diffusion-controlled electrochemical processes, the electroactive surface area (Aea) of electrodes can be calculated using the Randles–Sevcik equation (Ip = 2.69 × 10^5^n^3/2^AeaC√D*v*) [32]. In this equation, Ip represents the peak current (A), n is the number of electrons received or given, Aea is the electroactive surface area (cm^2^), C is the concentration of the solution (mol/cm^3^), D the diffusion coefficient (cm^2^/s), and ν (V/s) is the scan rate. The Aea values of SiO_2_NP@MWCNT-COOH-modified SPCEs were calculated as 0.2228 cm^2^ (1 LBL), 0.1874 cm^2^ (2 LBL), and 0.1893 cm^2^ (3 LBL). The highest Aea value was obtained with the electrode prepared using 1 LBL of SiO_2_NP@MWCNT-COOH. In the electrodes modified with 2 LBL and 3 LBL of SiO_2_NP@MWCNT-COOH, a thicker layer was formed compared to 1 LBL and the Aea values decreased. This decrease may be attributed to the thick layer formed on the surface, which hindered electron transfer. Based on these results, the highest conductivity was obtained with the SPCE/SiO_2_NP@MWCNT-COOH (1 LBL) electrode, indicating that the optimum amount of SiO_2_NP@MWCNT-COOH is 1 LBL.

### 3.2. Morphological and chemical characterization of modified electrodes

**Figure 1:**
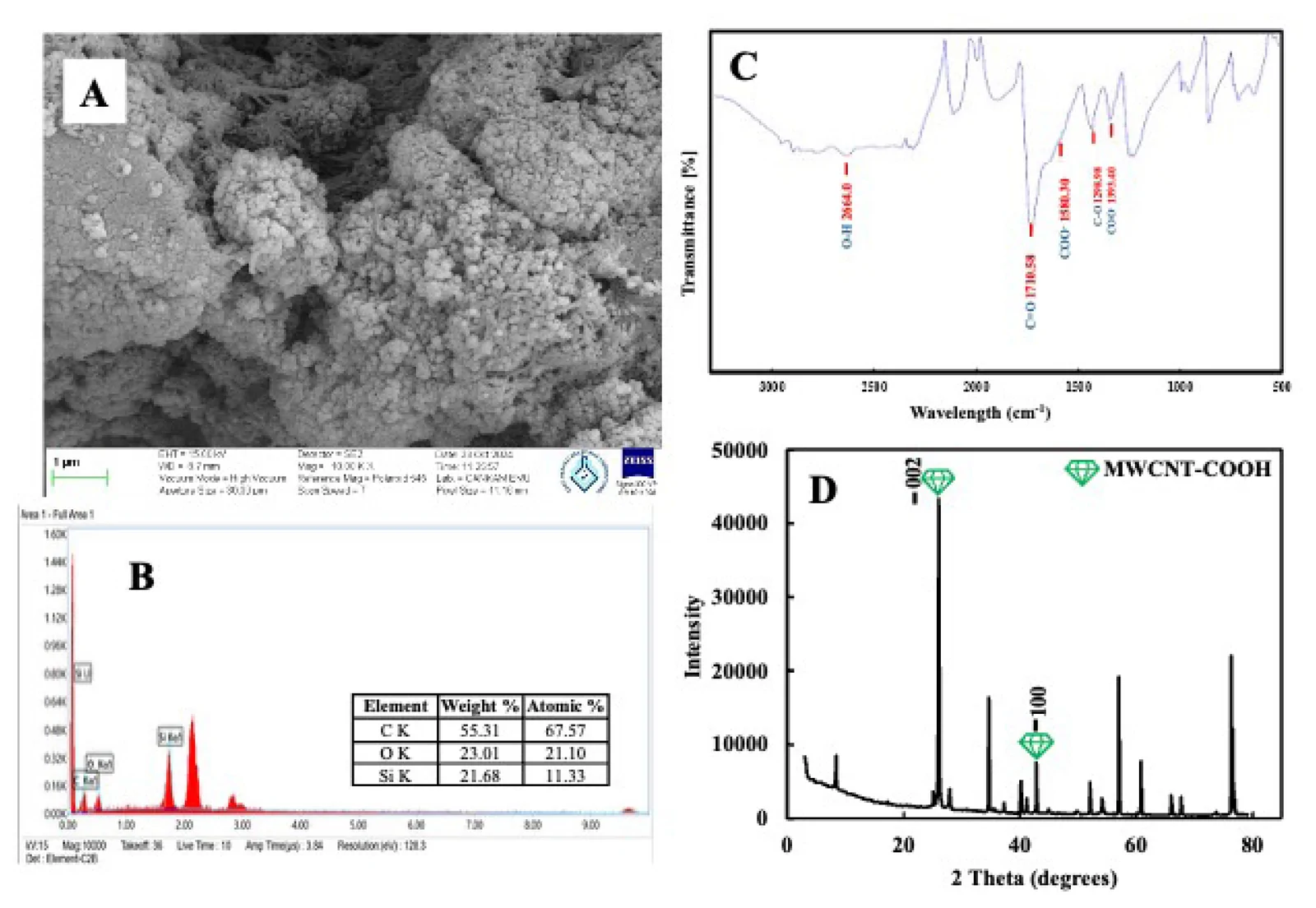
SEM images (A), EDX spectra (B), FTIR spectra (C), and XRD spectra (D) of SPCE/SiO_2_NP@MWCNT-COOH.

Morphological characterization of SPCE/SiO_2_NP@MWCNT-COOH electrodes was performed using SEM and chemical characterization was performed using EDX, FTIR, and XRD, respectively, as shown in Figures 1A–1D. In Figure 1A, the filamentous appearance of numerous spherical SiO_2_NPs and scattered MWCNT-COOH is evidence of the presence of SiO_2_NPs and MWCNT-COOH [33]. In Figure 1B, Si is observed in the EDX spectrum, confirming the presence of SiO_2_NPs on the electrode [34].

In the FTIR spectrum shown in Figure 1C, the broad absorption band at 2664.80 cm^−1^ is attributed to the O–H stretching vibration of the carboxylic acid (−COOH) groups linked by hydrogen bonds. The absorption peak at 1710.58 cm^−1^ corresponds to the C=O stretching vibration of the carboxyl group, while the bands at 1393.40 and 1298.98 cm^−1^ are assigned to the characteristic vibrations of the carboxyl functional groups, including the asymmetric/symmetric COO^−^ and C–O stretching vibrations. The band at 1580.30 cm^−1^ is the skeletal vibration of the aromatic/graphitic carbon bonds in the MWCNT structure [35].

In the XRD spectrum shown in Figure 1D, the sharp diffraction peak observed at 25.98° was successfully assigned to the characteristic (002) crystal plane (JCPDS No. 41-1487) of the MWCNT-COOH structure [36]. The other characteristic reflection peak from the graphene layers of the carbon nanotube structure was observed for the (100) plane at ~43.1° (2θ) [36]. On the other hand, due to the amorphous nature of SiO_2_NP in the composite structure, a broad distribution (halo) occurred in the spectrum in the range of 2θ = ~20°–23°; this wide amorphous base partially overlapped with the peak of the MWCNT at 25.98°, leading to an asymmetrical broadening at the peak base. Since the amorphous silica phase is dominant in the structure, no sharp crystal reflection peak belonging to SiO_2_NP was observed [37,38]. The simultaneous observation of characteristic diffraction peaks of both MWCNT-COOH and amorphous SiO_2_ in the XRD spectra, along with the identification of −COOH and Si–OH functional groups in FTIR analysis, confirmed the successful formation of a hybrid nanocomposite structure. Furthermore, the preservation of the characteristic structural properties of both components indicated that advantages such as the high electrical conductivity of MWCNT-COOH and the large surface area of SiO_2_NPs were largely retained within the composite structure. The −COOH and Si–OH groups detected in the FTIR spectrum highlight the formation of hydrogen-bond interactions between the two nanomaterials. These intermolecular interactions are considered to directly contribute to the development of a more stable and homogeneously distributed composite morphology.

### 3.3. Electrochemical performance of SPCE/MWCNT-COOH@SiO_2_NP

**Figure 2:**
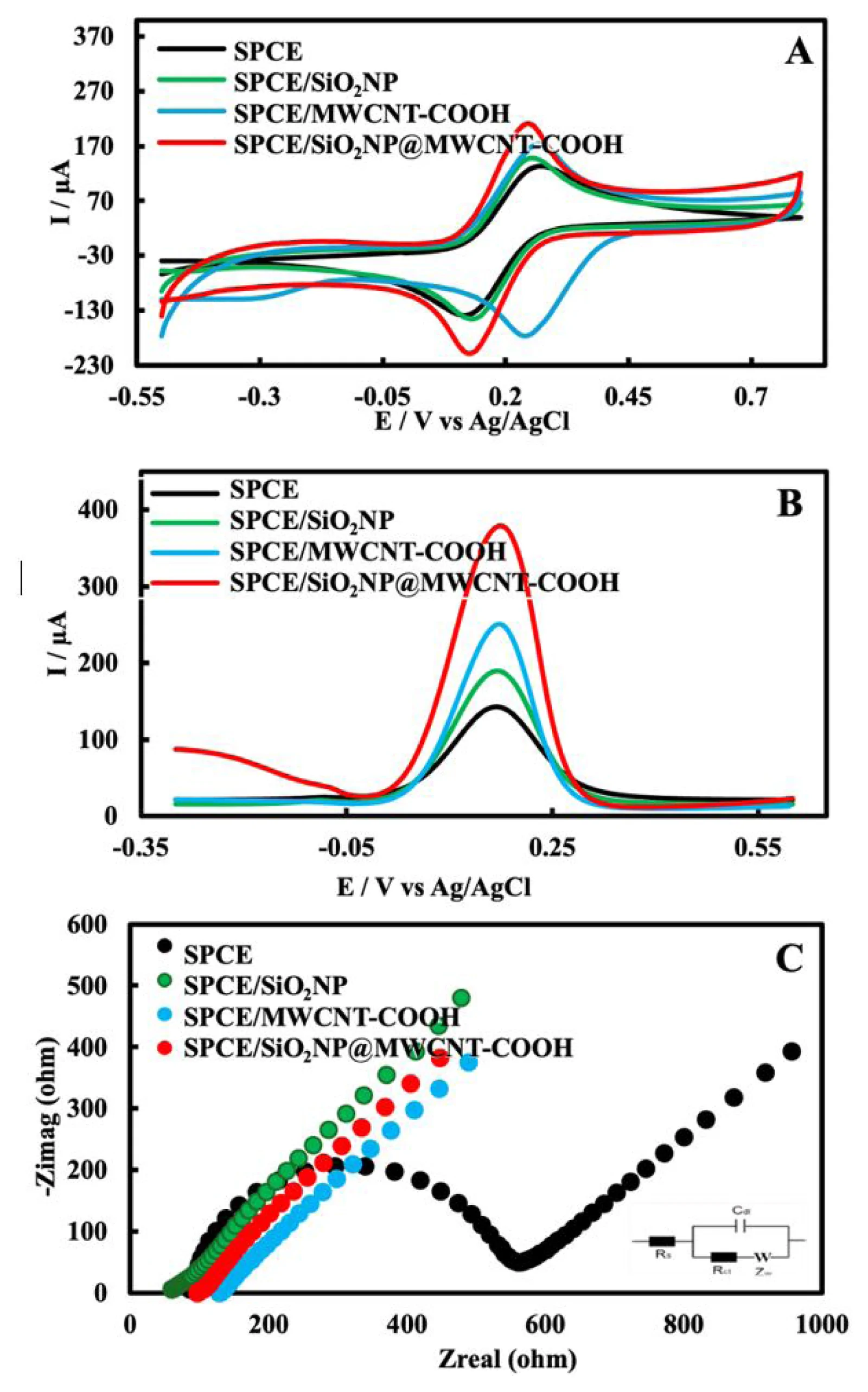
CV (A), DPV (B), and EIS (C) results for SPCE, SPCE/SiO_2_NP, SPCE/MWCNT-COOH, and SPCE/SiO_2_NP@MWCNT-COOH electrodes.

To investigate the electrochemical performance of the NPs, SPCEs were modified with SiO_2_NP, MWCNT-COOH, and SiO_2_NP@MWCNT-COOH and CV, DPV, and EIS measurements were performed in a redox probe solution. The CV, DPV, and EIS results are given in Figures 2A–2C, while Ipa_avg_ and Rct_avg_ are given in Table 1. According to Figure 2 and Table 1, Ipa_avg_ values increased and Rct_avg_ values decreased with SiO_2_NP modification compared to plain SPCEs. This result shows that SiO_2_NP modification improved the electrochemical response by increasing the electroactive surface area of the electrode. The increase in Ipa_avg_ values and the decrease in Rct_avg_ were more pronounced in the SPCE/MWCNT-COOH step because the carboxyl functional groups on the sp^2^ carbons in MWCNT-COOH have hydrophobic sidewalls and a π-conjugated structure. The highest electrochemical performance was observed for the electrode using SiO_2_NP and MWCNT-COOH together (SPCE/SiO_2_NP@MWCNT-COOH). The increase in peak currents and decrease in resistance occurred due to the formation of strong hydrogen bonds between the carboxyl groups in MWCNT-COOH and the silanol groups (≡Si–OH) on the SiO_2_NP surface, the integration of SiO_2_NP into the MWCNT-COOH networks, and the formation of a more stable, dispersed composite. Thanks to this stable structure, electron transfer from the electrode surface occurred more easily and quickly. Based on these results, the best-performing SPCE/SiO_2_NP@MWCNT-COOH was used as the interface in the development of the EP-NEP sensor. The superior electrochemical performance of the SPCE/SiO_2_NP@MWCNT-COOH electrode compared to individual components reveals a strong synergistic interaction between SiO_2_NP and MWCNT-COOH. The observed increase in peak current and the significant decrease in charge-transfer resistance (Rct) is fully consistent with the FTIR and XRD findings presented in Section 3.2. This demonstrates that hydrogen bonds and successful structural integration between the components significantly facilitated electron transfer at the electrode surface.

### 3.4. Electrochemical behavior of EP and NEP on the modified electrode

**Figure 3:**
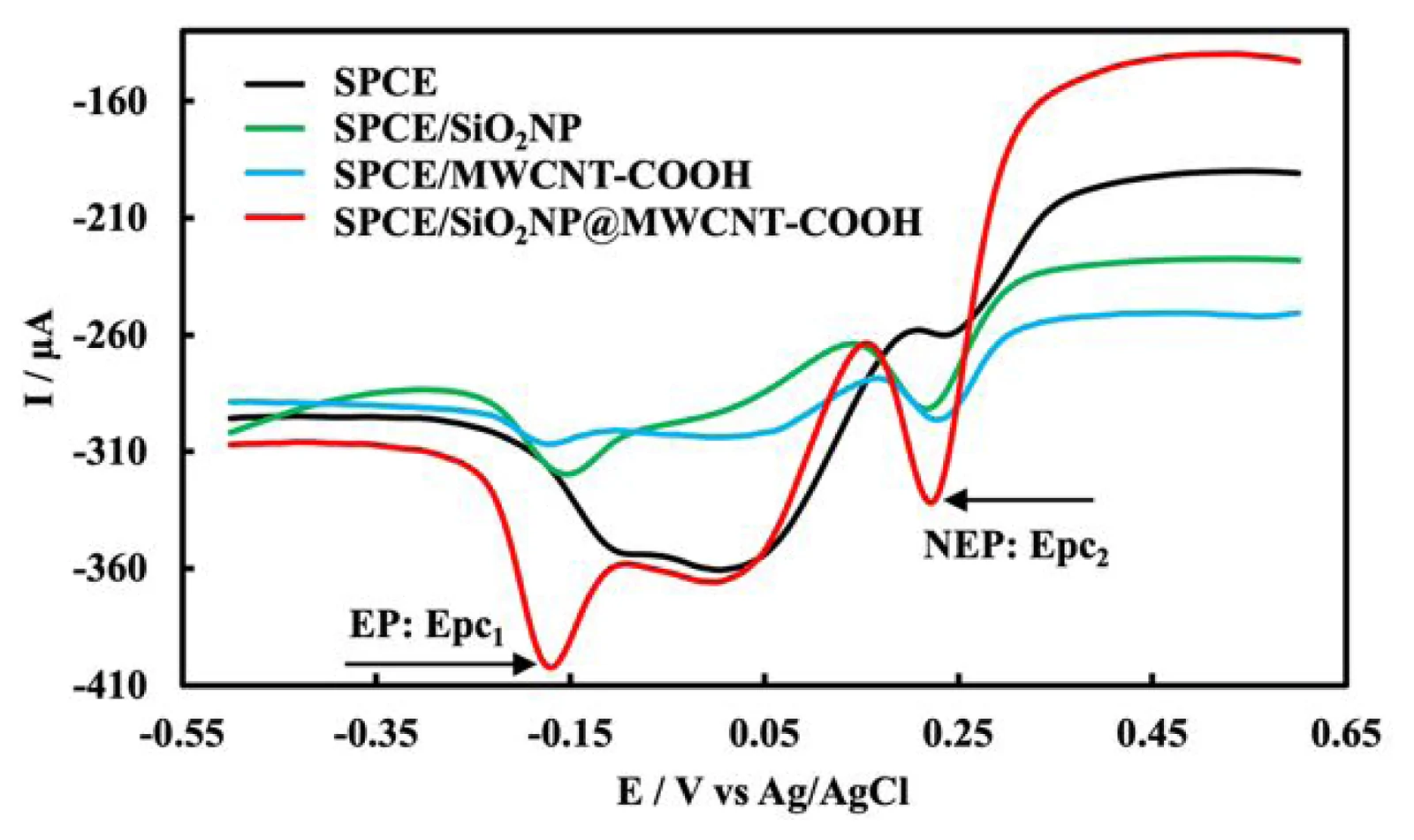
SWV results for SPCE, SPCE/SiO_2_NP, SPCE/MWCNT-COOH, and SPCE/SiO_2_NP@MWCNT-COOH electrodes in 50 μM EP-NEP solution (50 mM ABS, pH 4.5).

To investigate the effect of SiO_2_NP and MWCNT-COOH on the electrochemical behavior of EP and NEP, F measurements (−0.5 V to +0.6 V) were performed on modified electrodes with different formulations in 50 μM EP-NEP solution prepared in 50 mM acetate buffer (ABS; pH 4.5). SWV values are given in Figure 3 and cathodic peak currents (Ipc_1,2_) and cathodic peak potentials (Epc_1,2_) are given in Table 2. For bare SPCEs, the response of EP and NEP was quite low (Ipc_1_ for EP: −6.08 μA, Ipc_2_ for NEP: −1.62 μA). However, for the SPCE/SiO_2_NP electrode, the EP and NEP signals increased and the peak potentials shifted to more negative values (Epc_1_ for EP: −0.140 V → −0.155 V, Epc_2_ for NEP: 0.425 V → 0.215 V). This signal increase occurred due to the interaction of EP and NEP and the silanol groups on SiO_2_NP electrodes, which accelerated electron transfer. Furthermore, SiO_2_NPs reduced the electroreduction potential of EP and NEP, thus exhibiting an electrocatalytic effect. For the SPCE/MWCNT-COOH electrode, peak currents increased compared to SPCEs and the EP potential shifted to negative values. For the electrode containing both SiO_2_NPs and MWCNT-COOH (SPCE/SiO_2_NP@MWCNT-COOH), the response of EP and NEP compared to SPCEs resulted in a 10-fold and 100-fold increase in peak currents, respectively. Peak potentials, however, were significantly lower (Epc_1_ for EP: −0.170 V, Epc_2_ for NEP: 0.220 V). The SPCE/SiO_2_NP@MWCNT-COOH electrode exhibited an electrocatalytic effect in the electroreduction of EP and NEP.

**Figure 4:**
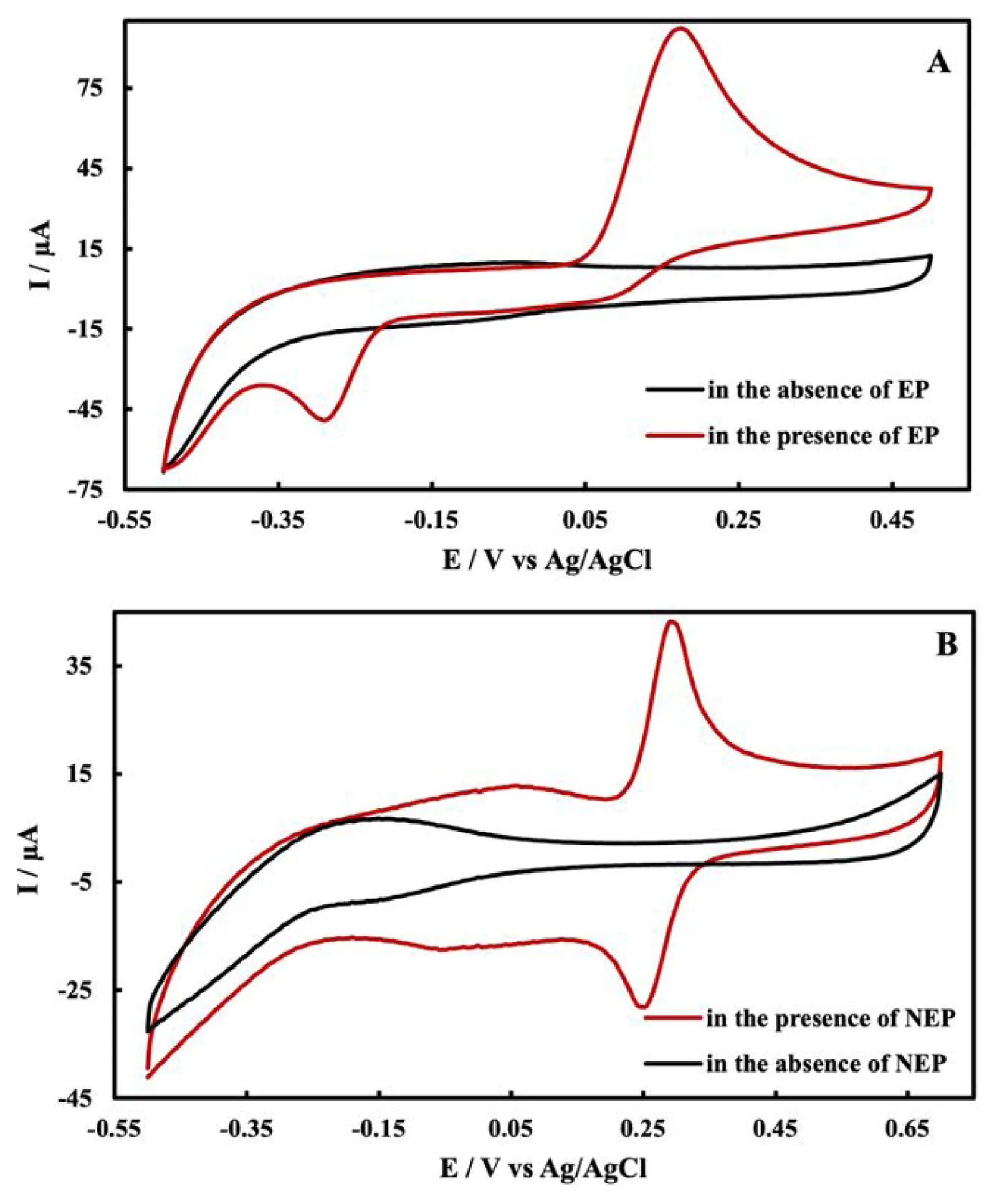
CV results for SPCE/SiO_2_NP@MWCNT-COOH with and without 1 mM EP (A) and 1 mM NEP (B).

To investigate the electrochemical behavior of EP and NEP and the electrocatalytic activity of the SPCE/SiO_2_NP@MWCNT-COOH electrode, CV was performed (−0.50 V to +0.70 V, 50 mV s^−1^) in the presence and absence of 1 mM EP and 1 mM NEP (50 mM ABS, pH 4.5, 0.1 M KCl) (Figures 4A and 4B). As seen in Figures 4A and 4B, no peaks were observed in the buffer solution in the absence of EP and NEP. According to Figure 4A, in the presence of 1 mM EP, the oxidation peak of EP was observed at 0.182 V and the reduction peak was observed around −0.296 V. In the literature, it has been reported that the electroreduction peak of EP at pH 4.9 is −0.160 V (Ec_2_) and the oxidation peaks are approximately 0.32 V (Ea_1_) and −0.135 V (Ea_2_), respectively [39]. According to Figure 4B, in the presence of 1 mM NEP, the oxidation peak of NEP was observed at 0.304 V and the reduction peak at around 0.256 V. In the literature, the electroreduction and oxidation peaks of NEP are reported to occur at approximately 0.216 and 0.217 V, respectively [40].

**Figure 5:**
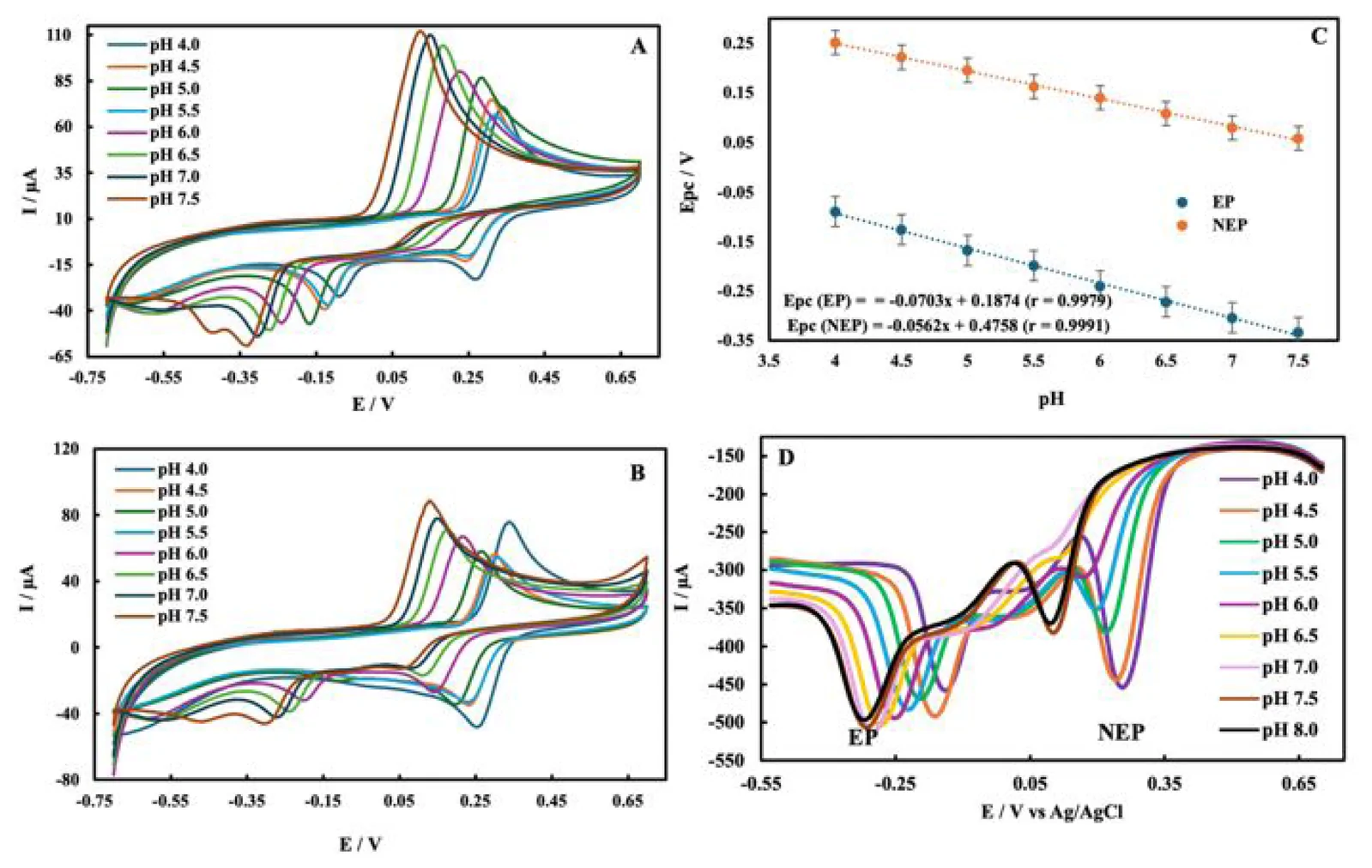
CV results for SPCE/SiO_2_NP@MWCNT-COOH in 1 mM EP (A) and 1 mM NEP (B) at various pH levels (at 50 mV s^−1^); graph of Epa versus pH (C); SWV results in 50 μM EP-NEP solution (D).

To investigate the effect of pH on the electroreduction of EP and NEP on the SPCE/SiO_2_NP@MWCNT-COOH electrode, EP and NEP solutions were prepared in acetate and phosphate buffers at various pH values (pH 4–7.5), and separate CV (1 mM EP and NEP) and simultaneous SWV (50 μM EP-NEP) measurements were taken. The CV results obtained at different pH values are given in Figures 5A and 5B, the Epc-pH graph in Figure 5C, and the SWV results in Figure 5D. In CV, as pH increased, the anodic and cathodic peak currents also increased, and the anodic and cathodic peak potentials shifted to negative values. The shift in Epa and Epc values occurred due to the pH dependence of electrooxidation. From the SWV results for the EP-NEP solution, as shown in Figure 5D, pH 4.5 was selected as the optimum pH because it yielded the best peak separation.

### 3.5. Analytical performance of individual and simultaneous EP and NEP sensors

To examine the analytical performance of the prepared sensors, separate and simultaneous determinations of EP and NEP were performed and their linear working ranges and repeatability were determined. In addition, reproducibility, shelf life, and selectivity studies of the sensor were also carried out for the simultaneous determination of EP and NEP. Separate and simultaneous determinations of EP and NEP sensors were performed using the SWV method with SPCE/SiO_2_NP@MWCNT-COOH electrodes in 50 μM ABS at pH 4.5.

**Figure 6:**
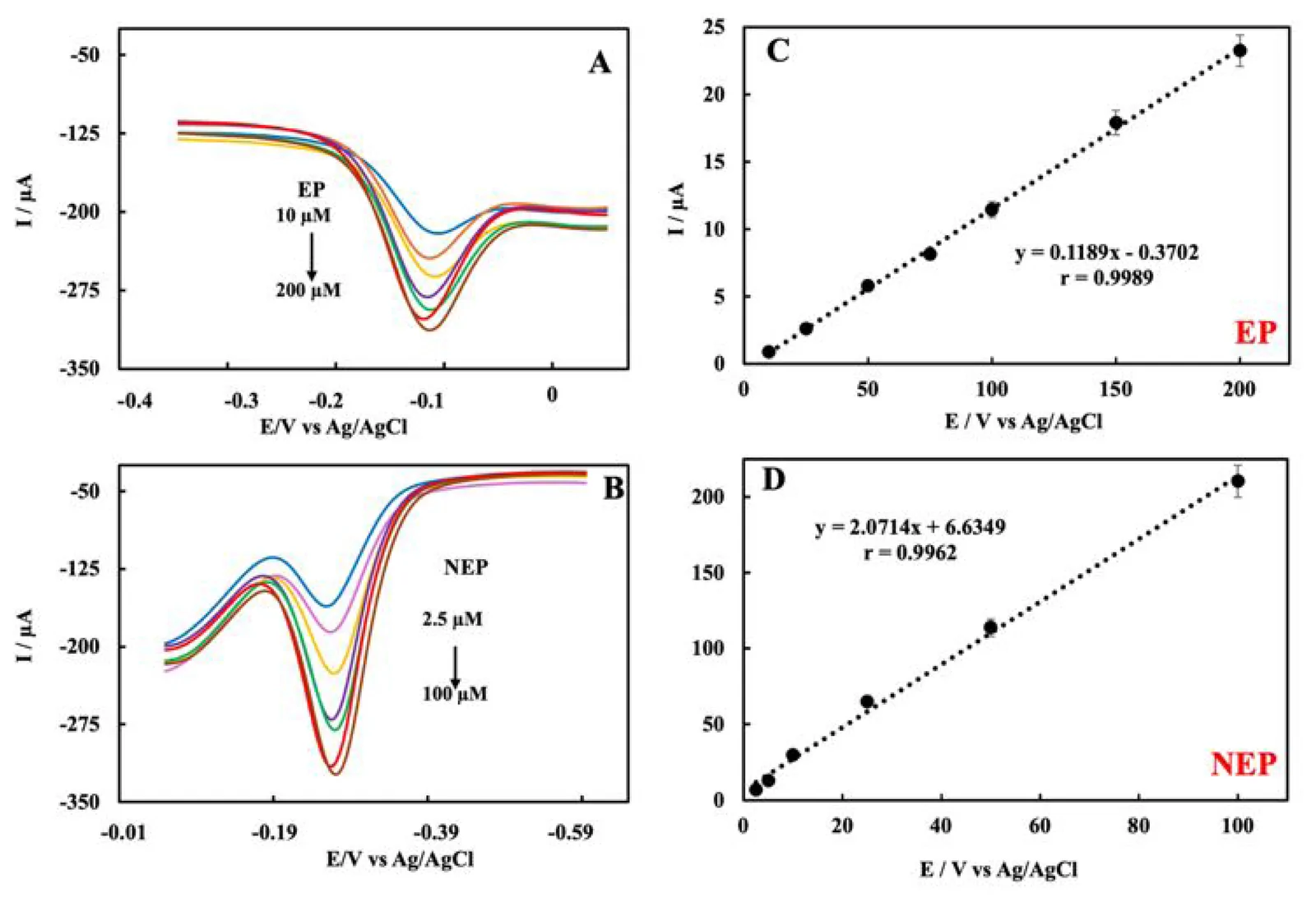
Individual determination of EP and NEP: SWV results for SPCE/SiO_2_NP@MWCNT-COOH at varying concentrations of EP (A) and NEP (B); calibration graphs for EP (C) and NEP (D).

For the individual determination of EP and NEP, the SWV results obtained at various EP (10–200 μM) and NEP (2.5–100 μM) concentrations are given in Figures 6A and 6B, respectively, and the Ipc-C graphs are given in Figures 6C and 6D, respectively. As seen in Figures 6A and 6B, the currents increase linearly with the concentrations of EP and NEP.

**Figure 7:**
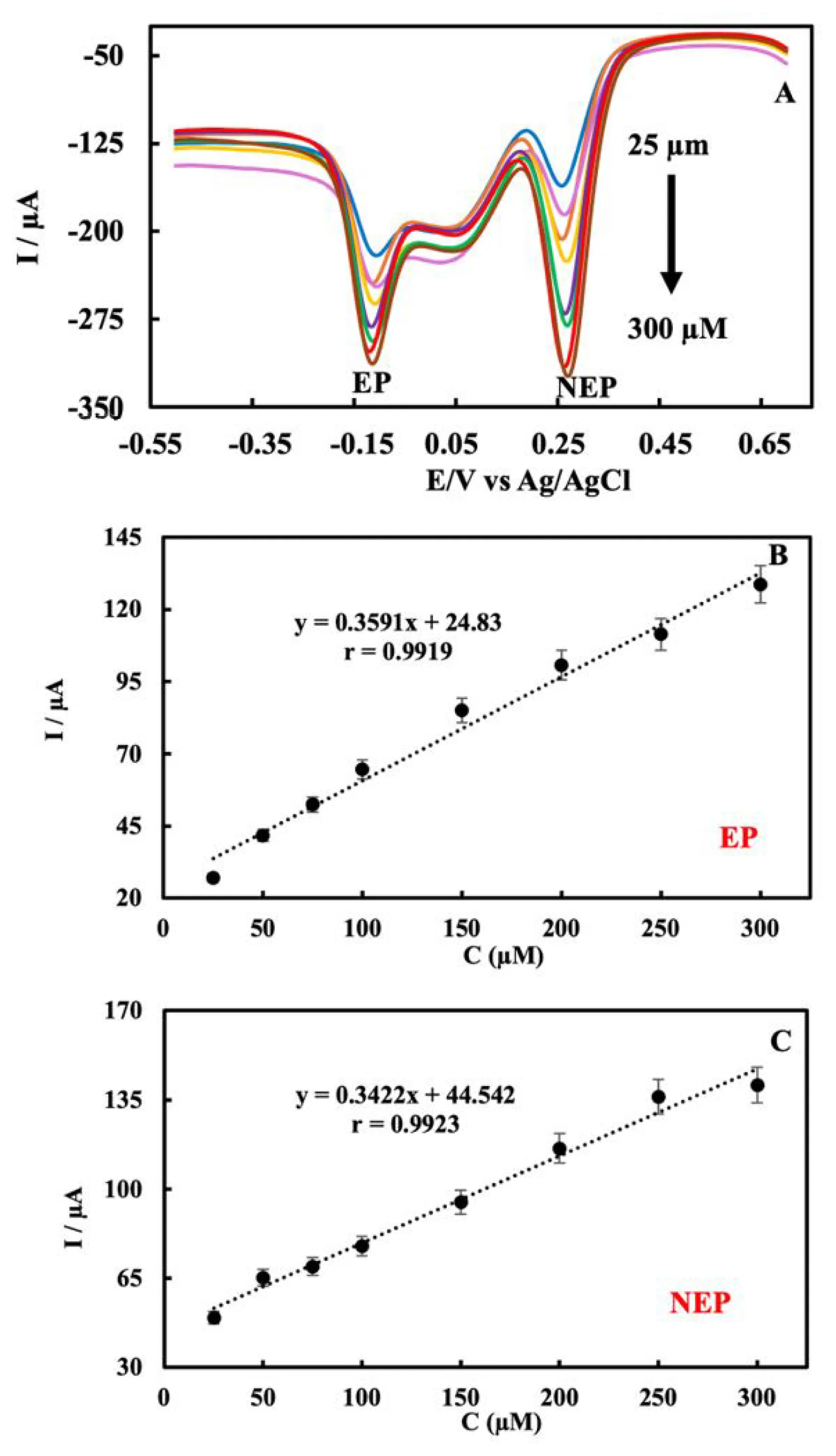
Simultaneous determination of EP and NEP: SWV results for SPCE/SiO_2_NP@MWCNT-COOH electrode at varying concentrations of mixture of EP-NEP (A); calibration graphs for EP (B) and NEP (C).

The linear regression equations obtained from the calibration curve were y = 0.1189x – 0.3702 (r = 0.9989) for EP and y = 2.0714x + 6.6349 (r = 0.9987) for NEP. The limit of detection (LOD) values were calculated to be 1.08 μM and 0.61 μM for EP and NEP, respectively (3 s/m). The SWV results obtained in the EP-NEP concentration range of 25–300 μM for the simultaneous determination of EP and NEP are given in Figure 7A and the Ipc-C graphs are given in Figures 7B and 7C. The electroreduction peak currents of EP and NEP increased with increasing concentrations. The linear regression equations obtained from the calibration curve were y = 0.3591x + 24.83 (r = 0.9919) for EP and y = 0.3422x + 44.542 (r = 0.9923) for NEP. The LOD values were calculated to be 13.43 and 16.61 μM for EP and NEP, respectively (3 s/m). Although EP and NEP exhibited well-resolved and completely separated peak potentials with a significant potential difference of ~400 mV (Figure 7A), a noticeable increase in LOD values was observed during their simultaneous determination (13.43 μM for EP and 16.61 μM for NEP) compared to individual measurements. This may be primarily attributed to the competitive adsorption and steric hindrance between EP and NEP molecules at the limited active sites (such as carboxylic acid groups) on the SPCE/SiO_2_NP@MWCNT-COOH surface. When both analytes coexisted in the electrochemical cell, their interactions during diffusion and adsorption limited the effective surface coverage of each species compared to their individual solutions. Additionally, the simultaneous electrochemical reactions of both analytes across the scanned potential range contributed to an elevated background charging current, thereby increasing baseline noise and shifting the limit of detection toward higher concentrations. In summary, this study demonstrates that EP and NEP can be determined at low concentrations, either individually or simultaneously, with high sensitivity and a wide linear range using SPCE/SiO_2_NP@MWCNT-COOH electrodes.

The levels of EP and NEP in healthy human blood serum are reported to range from 40 to 90 μM, respectively [41]. EP and NEP levels outside these values are used to diagnose various diseases. Simultaneous electrochemical determination of EP and NEP with the SPCE/SiO_2_NP@MWCNT-COOH electrode was performed quickly and practically with high sensitivity and a wide linear range at low concentrations of 10 and 2.5 μM, respectively. Furthermore, the sensor’s high sensitivity and electroactive surface area were attributed to increased electronic conductivity and rapid electron transfer. A comparison of the individual and simultaneous EP and NEP sensors developed in recent years with the separate and simultaneous EP and NEP sensors we developed in this study is given in Table 3.

The analytical performance of our developed SPCE/SiO_2_NP@MWCNT-COOH sensor compared to EP/NEP platforms reported in the literature demonstrates the high potential of this hybrid nanocomposite architecture for the simultaneous determination of these analytes with quantitative accuracy. The originality of this approach lies in the synergistic effect of the material’s structural advantages on the electroanalytical behavior of EP and NEP, and in the strong performance parameters achieved. Accordingly, the 390-mV peak separation obtained in our study (Table 2) is among the highest values reported in the literature for the simultaneous determination of EP and NEP. This value, which ensures the selective and interference-free determination of two structurally similar neurotransmitters, dopamine (DA) and serotonin (5-HT), by preventing the overlap of oxidation signals, is superior compared to the peak separations in the range of 200–350 mV reported for sensors using CTAB-SnO_2_ nanoparticles [10] and some MOF-based materials [26]. Furthermore, the sensitivity of our sensor is clear from increases of approximately 10.8-fold and 67-fold in EP and NEP peak currents, respectively, compared to the bare SPCE. This powerful signal amplification can compete with or surpass many existing sensors in terms of detection limits (1.08 μM for EP and 0.61 μM for NEP), as detailed in Table 3. Compared to other studies, our developed sensor exhibits a fairly wide linear dynamic range extending up to 300 μM. Beyond simple numerical limitations, the proposed SPCE/SiO_2_NP@MWCNT-COOH platform significantly outperforms previous studies in terms of ease of fabrication, analysis time, superior neuroselectivity, and long shelf life. While conventional glassy carbon or pyrolytic graphite electrodes require laborious, unrepeatable mechanical surface polishing before modification, the disposable, low-cost SPCEs used in this study offer superior performance and user-friendly operation without the need for surface pretreatment. Furthermore, nanomaterials can be modified onto the SPCE quickly and easily without the need for complex chemical processes.

The repeatability of the prepared sensors was tested by individual and simultaneous SWV measurements for 50 μM EP and 50 μM NEP solutions. The relative standard deviation (RSD) values for individual determinations of EP and NEP were calculated as 4.60% (EP, n = 9) and 2.71% (NEP, n = 9), respectively, while for simultaneous determinations they were calculated as 4.28% (EP, n = 5) and 4.52% (NEP, n = 5). These results indicate that the prepared sensors can be used to perform up to 9 replicates of individual EP and NEP analyses and up to 5 replicates of simultaneous EP-NEP analyses.

**Figure 8:**
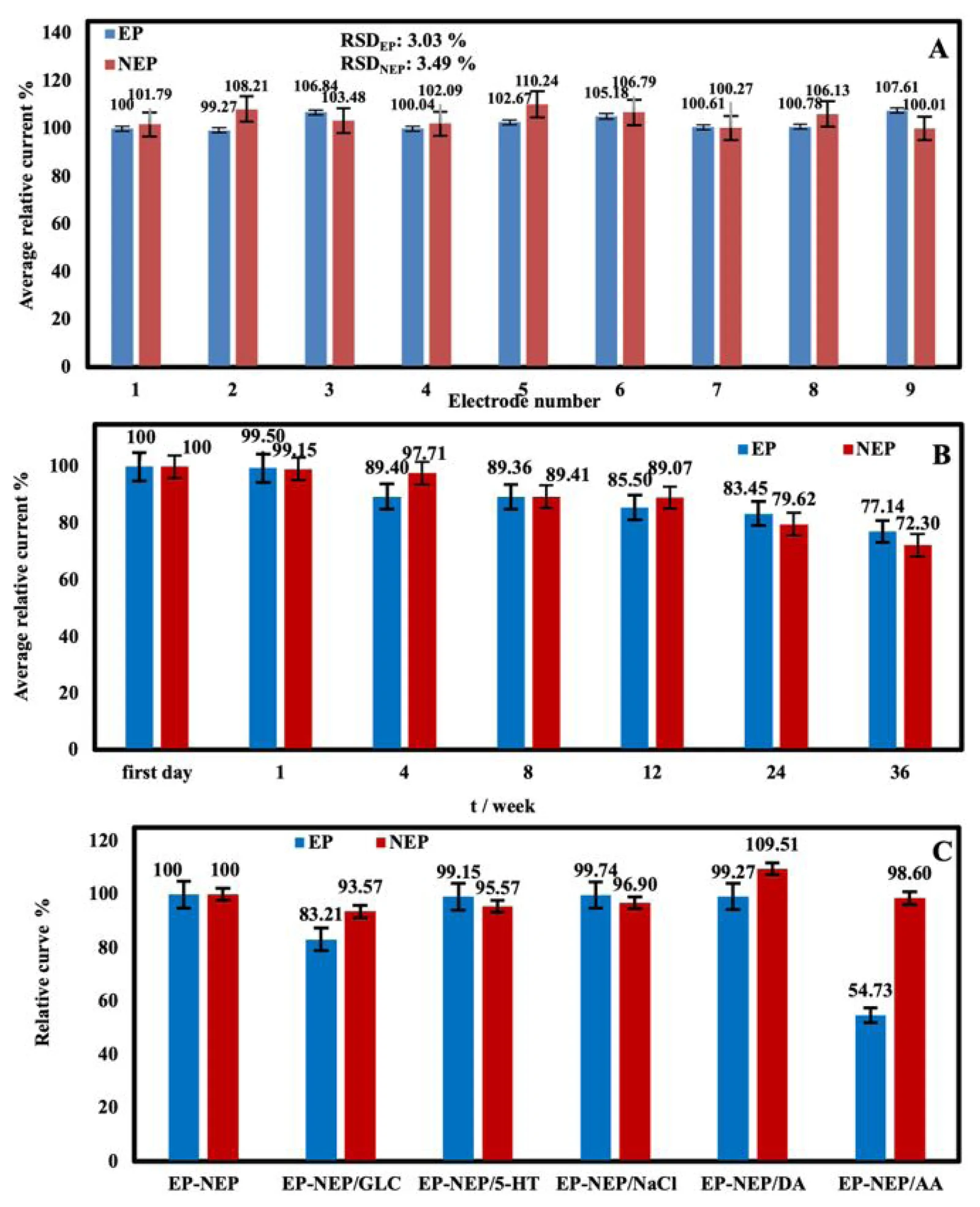
Reproducibility study (A); shelf-life study (B); selectivity study (C).

To investigate the reproducibility of the sensor in the simultaneous determination of EP-NEP, 9 different SPCE/SiO_2_NP@MWCNT-COOH electrodes were prepared at separate time points and SWV measurements were performed with the EP-NEP mixture (both at 50 μM). The %Ipc-electrode number graph is given in Figure 8A and the RSD values for EP and NEP were calculated as 3.03% and 3.49%, respectively.

To assess the long-term stability and shelf life of the fabricated EP-NEP sensor, SPCE/SiO_2_NP@MWCNT-COOH electrodes were placed in petri dishes to minimize atmospheric moisture absorption and kept in a refrigerator at 4 °C in the dark. Stability measurements were performed in triplicate using the SWV method in a mixture containing 50 μM EP and NEP on the initial day and at weeks 1, 4, 8, 12, 24, and 36. As illustrated by the average relative current percentages over time in Figure 8B, the sensor response decreased by only 10.6% for EP and 2.30% for NEP at the 4th week. Remarkably, even at the end of the 36th week, the current signal loss was limited to 22.86% for EP and 27.7% for NEP. These findings demonstrate that the synergetic modification with MWCNT-COOH and SiO_2_ nanomaterials significantly enhanced the long-term stability, allowing the EP-NEP sensor to retain over 70% of its initial response after 36 weeks.

To test the sensor’s selectivity, EP and NEP were simultaneously measured in the presence of glucose (GLC), ascorbic acid (AA), 5-HT, DA, and sodium (Na^+^), each at 100 μM. The relative current percentage interference agent graphs obtained from SWV are given in Figure 8C. Remarkably, the sensor exhibited outstanding tolerance toward 5-HT and DA. Although DA and 5-HT are known to exhibit strongly overlapping electrochemical reaction potentials with EP and NEP on conventional surfaces, the proposed platform maintained excellent and stable relative current responses in their presence. This clear resolution demonstrates the nanostructured framework’s electrocatalytic resolution and antiinterference capability against chemically and electrochemically similar neurotransmitters. Furthermore, the addition of GLC and NaCl resulted in minor or negligible variations within acceptable limits. In contrast, AA induced a profound and selective suppression exclusively on the EP reduction peak (dropping to 54.73%), while leaving the NEP signal completely unaffected (98.60%). This distinct behavior is governed by an electrochemical-chemical pathway in which the secondary amine group of EP accelerates the cyclization of electrogenerated epinephrine quinone, thereby causing rapid chemical reduction by AA in the diffusion layer before voltametric measurement. The primary amine in NEP yields a more stable quinone that resists this transient chemical depletion. Consequently, the proposed architecture offers a highly reliable, high-resolution, and predictable selectivity model for catecholamine determination.

### 3.6. Simultaneous analysis of EP-NEP in commercial blood serum

Simultaneous analyses of EP-NEP in commercial human blood serum (H6914, Sigma-Aldrich, St. Louis, MO, USA) were performed by SWV measurements using SPCE/SiO_2_NP@MWCNT-COOH electrodes. Commercial human blood serum (H6914) diluted to a ratio of 1:100 (PBS, pH 7.4) was spiked with different concentrations of EP and NEP solutions (50 μM, 100 μM, and 200 μM), and SWV measurements were performed [42–44]. The results of the simultaneous analysis of EP and NEP in commercial human blood serum are shown in Table 4. The analysis confirmed that high recoveries were obtained for both EP and NEP, and the error values were approximately 5.5% or less. In this study, the simultaneous analysis of EP and NEP in commercial human blood serum was performed practically and quickly within 1–2 min using the SWV method, without requiring lengthy procedures. Although the current protocol relies on prediluted samples to minimize matrix effects, the analytical efficiency and user-friendly nature of this single-use platform indicate considerable promise for future research toward point-of-care testing applications, provided that it undergoes further comprehensive clinical optimization.

## 4. Conclusion

This study investigated the effect of electrodes prepared with different combinations of SiO_2_NP and MWCNT-COOH nanomaterials on the electrochemical behavior of EP and NEP. The SiO_2_NP@MWCNT-COOH nanocomposite showed a strong synergistic effect on the electroreduction of EP-NEP, indicating both electronic and electrocatalytic effects. Surface analyses (SEM-EDX, XRD, FTIR) confirmed the preparation of the SPCE/SiO_2_NP@MWCNT-COOH sensor. Using the developed sensors, individual and simultaneous determinations of NE and NEP were performed with low detection limits, wide linear ranges, and good reproducibility. The sensor’s good selectivity was confirmed by the absence of significant changes in the EP and NEP signals, even in the presence of potential interfering agents. The storage stability of the EP-NEP sensor was evaluated over 36 weeks, yielding good results. Simultaneous analysis of EP and NEP in commercial human blood serum samples showed that the sensor provides reliable results with high recovery (>95%) and low error rates (<5%) in real matrices. These results demonstrate that the prepared sensors are unaffected by other substances, even in complex samples with high matrix effects such as blood serum.

Consequently, the SPCE/SiO_2_NP@MWCNT-COOH sensor developed in this study demonstrates excellent analytical performance for the simultaneous determination of EP-NEP, characterized by low detection limits, good selectivity, and high reproducibility. Although the real-sample validation in this work was confined to preliminary spike-and-recovery experiments in serum, the obtained results confirm the fundamental reliability of the proposed method. To fully establish its clinical utility, future studies will focus on comprehensive testing with real patient cohorts. The preliminary results obtained with this practical, low-cost sensor provide robust proof-of-concept, paving the way for future point-of-care and clinical diagnostic platforms.

## Supplementary material

### 1. Experimental

#### 1.1. Chemicals

Potassium hexacyanoferrate (K_3_Fe(CN)_6_), potassium hexacyanoferrite (K_4_Fe(CN)_6_, sodium acetate (CH_3_COONa), acetic acid (CH_3_COOH), human serum (from male AB clotted whole blood, H6914), dopamine (DA), serotonin (5-HT), glucose (GLC), epinephrine (EN), ascorbic acid (AA) norepinephrine (NEP) was obtained from Sigma-Aldrich (USA). L-, silica dioxide nanoparticle (SiO_2_NP), carboxyl functionalized form of multi-walled carbon nanotubes (MWCNT-COOH), potassium chloride (KCl), potassium dihydrogen phosphate (KH_2_PO_4_), potassium hydrogen phosphate (K_2_HPO_4_), and hydrochloric acid (HCl) were obtained from Merck. CH_3_COONa and CH_3_COOH were used to prepare Acetate Buffer (ABS). K_2_HPO_4_, KH_2_PO_4_, and KCl were used to prepare phosphate buffer solution (PBS). All of the solutions used were prepared with ultrapure water (Millipore,18MΩ cm).

#### 1.2. Instruments

The voltammetric analysis was performed using a DropSens μStat 4000P Multi Potentiostat from Methrom DropSens (Oviedo, Spain) and operated by a PC running DropView 800 software. Gamry Potentiostate/Galvanostate, Reference 1010E (Gamry Instruments, Warminster, USA), electrochemical impedance spectroscopy (EIS) measurements were performed on a computer running EChem Analyst. The SPCEs were purchased by Methrom DropSens (Oviedo, Spain, DRX110). SPCE consists of a combined three-electrode system with a working (WE) and a counter electrode of carbon and a reference electrode of silver. All solid chemicals were weighed using a Radwag AS220/C/2 electronic precision balance with a sensitivity of 0.01 mg. A WiseStir MSH-20D heated stirrer and an IKA MS 3 digital vortex device were used to prepare the solutions. Solutions were prepared with water supplied by a Millipore Milli-Q Direct Q-3 ultrapure water device. The pH values required for the preparation of the buffer solutions were provided by a Mettler-Toledo pH meter. Standard buffer solutions were used for calibration, and the pH meter was prepared for measurement. For chemical and morphological characterizations, Perkin Elmer Fourier transform infrared (FT-IR), Panalytical Empyrean X-ray diffraction (XRD), and ZEISS Gemini 1 Field Emission Scanning Electron Microscope (FE-SEM) devices were used.

#### 1.3. Electrochemical measurements

Electrochemical characterizations were carried out with cyclic voltammetry (CV), differential pulse voltammetry (DPV), and EIS in redox probe solution (5 mM redox probe solution: K_3_Fe(CN)_6_/K_4_Fe(CN)_6_ in 1 M KCl). EIS measurements were carried out at a 50.000 Hz - 0.05 Hz frequency range. The following parameters were used for CV measurements: potential ranges of −0.5V to +0.8V at 50 mV s^−1^. DPV measurement parameters were as follows: pulse potential, 70 mV; pulse time, 0.1 s; step potential, 5 mV; and scan rate, 5 mV s^−1^. Analytical characterizations (linear range, repeatability, etc.) of EP and NEP were performed using the SWV method (from −0.5 V to +0.6 V).

Supplementary Figure S CVs of the different SiO_2_NP@MWCNT-COOH layers (1 LBL, 2 LBL and 3 LBL) SPCE/SiO_2_NP@MWCNT-COOH in 5 mM redox probe solution at different scan rates (10–25–50–75–100–125 mV s-1) Ipa-v1/2 graphs (A, B, C), Ipa-v1/2 graphs (D, E, F).

## Figures and Tables

**Table 1:** Ip_avg_ and Rct_avg_ values obtained from the CV, DPV, and EIS analysis of modified electrodes in redox probe solution.

	CV	DPV	EIS
Electrode formulation	Ipa_avg_ (μA)	Ipa_avg_ (μA)	Rct_avg_ (ohm)
SPCE	134.73	143.55	377.10
SPCE/SiO_2_NP	145.99	172.65	240.40
SPCE/MWCNT-COOH	159.79	221.73	229.20
SPCE/SiO_2_NP@MWCNT-COOH	185.85	350.51	89.12

**Table 2:** Values of Ep and Ip obtained from SWV of the SPCE/SiO_2_NP@MWCNT-COOH electrodes at each preparation step in a solution mixture of EP and NEP (each at 50 μM).

	EP		NEP	
Electrode formulation	Ipc_1_ (μA)	Epc_1_ (V)	Ipc_2_ (μA)	Epc_2_ (V)
SPCE	−6.08	−0.140	−1.62	0.425
SPCE/SiO_2_NP	−28.35	−0.155	−39.5	0.215
SPCE/MWCNT-COOH	−9.54	−0.170	−26.88	0.230
SPCE/SiO_2_NP@MWCNT-COOH	−65.43	−0.170	−108.46	0.220

**Table 3:** Performance comparisons of individual and simultaneous EP and NEP sensors in the literature and the sensor developed in our study.

Electrochemical EP sensors				
Electrode formulation	Method	Linear range	LOD	Ref.
CPE/PA/CNT	DPV	2.0–45.0 μM	0.25 μM	[13]
GCE/MWCNT/Capsaicin	CA	50–1150 μM	7.2 μM	[14]
GCE/MWCNT/MoS_2_	CV	9.9–137.9 μM	3.0 μM	[15]
GCE/MWCNT/ZnO	DPV	0.4–2.4 μM	0.016 μM	[16]
GR/BDD	CV	10–200 μM	2.7 μM	[45]
GCE/CRGO	DPV	10–300 μM 400–1300 μM	1.6 μM	[17]
GCE/MGO	CV	100–600 μM	0.13 μM	[46]
ITO/GMA	DPV	1–60 μM	0.0035 μM	[47]
ITO/GF@NiMoS_4_NWAs	DPV	0–60 μM	0.02 μM	[18]
flexible microelectrode/Cu/CuO-VG	DPV	0.5–100 μM	0.13 μM	[48]
CPE/CNF	DPV	0.1–150 μM μM	0.03 μM	[49]
GCE/CNF-AuNP	SWV	50 μM to 1 mM	1.70 μM	[50]
GCE/CNF-CoMnZIF	DPV	5–1000 μM	7.20 μM	[51]
GCE/g-C_3_N_4_/NCDS/CQD	CV	1 pM to 1 nM	0.30 pM	[52]
GCE/CuO/CQD	CV	10–100 μM	15.99 μM	[19]
Electrochemical NEP sensors				
Electrode formulation	Method	Linear range	LOD	Ref.
SPCE/3-APBA	CV	0.1–5 pM	0.03 pM	[20]
GCE/NiO-RGO	CA	20 nM–14 μM 14 μM–80 μM	0.005 μM	[21]
SPCE/TiO_2_/MXene-PVA/GO	CA	0.01–1 μM 1–60 μM	0.008 μM	[53]
GCE/poly-(TERE-AHNSA)	DPV	100 nM –1600 μM 5.0 nM–100 μM	2.08 nM	[40]
MCPE/ MnCr_2_O_4_	CV	0.3–4.5 μM	0.034 μM	[54]
CPE/SiTi/AuNP	SWV	20–180 μM	0.35 μM	[55]
GCE/CB/NiFe LDH	DPV	0.1–100 μM	3 nM	[56]
GCE/Pd-ERGO	DPV	0.5–100 μM	0.05 μM	[57]
GCE/AMO-MWCNT-NP	DPV	10–100 μM	4.04 nM	[58]
GCE/H-MOF@PPy-40	DPV	0.1–10 μM 10–1000 μM	0.033 μM	[22]
SPCE/D-SWCNTs	DPV	0.10–2 μM	0.062 μM	[59]
CPE /Y-Ni-MOFs/IL	DPV	0.05–700 μM	0.025 μM	[23]
Electrochemical EP-NEP Sensors				
Co-Ni/BTC/4,4′-BiPy-MOFs		10–50 μM	EP: 0.724 μM NEP: 0.815 μM	[26]
PGE-COOH	SWV	EP: 2–100 μM NEP: 2–250 μM	EP: 1.40 μM NEP: 1.46 μM	[24]
GCE/SnO2/CTAB	SWV	EP: 0.1–250 μM NEP: 0.1–300 μM	EP: 10 nM NEP: 6 nM	[10]
EPPGE/MWNT	SWV	0.5–100 nM	EP: 0.15 × 10^−9^ M NEP: 0.90 × 10^−10^ M	[25]
SPCE/SiO_2_NP@MWCNT-COOH	SWV	EP–NEP: 25–300 μM EP: 10–200 μM NEP: 2.5–100 μM	EP: 13.43μM, NEP: 16.61 μM EP: 1.08 μM NEP: 0.61 μM	This work

3-APBA: 3-Aminophenylboronic acid

AMO: Silver manganese oxide

AuNP: Gold nanoparticle

BDD: Boron-doped diamond bimetallic Co-Ni/BTC/4,4′-BiPy

CA: Chronoamperometry

CB: Carbon black

CNF: Carbon nanofiber

CNT: Carbon nanotube

CPE: Carbon paste electrode

CRGO: Chemically reduced graphene oxide

CTAB: Cetyltrimethylammonium bromide

CV: Cyclic voltammetry

DPV: Differential pulse voltammetry

D-SWCNTs: Debundled single-walled carbon nanotubes

EPPGE: Edge plane pyrolytic graphite electrode

g-C_3_N_4_/NCDS: Graphitic carbon nitride/N-doped carbon dot composite

GCE: Glassy carbon electrode

GMA: Reduced graphene oxide/Ti_3_C_2_T_x_ MXene GR: Graphene

H-MOF@PPy-40: Hollow core-shell conductive composite materials

IL: Ionic liquid

ITO: Indium tin oxide

MCPE: Modified carbon paste electrode

MGO: Melamine modified graphene oxide

MnCr_2_O_4_: Manganese(II) chromate

MoS_2_: Molybdenum disulfide

MWCNT: Multiwalled carbon nanotube

MWNT: Multiwalled nanotube

NiFe LDH: NiFe-layered double hydroxide nanosheets

NiMoS_4_NWAs: Nickel molybdenum sulfide nanowire arrays

NiO: Honeycomb-like nickel oxide

NP: Nanoparticle

PAG: Polyasparagine

Pd: Palladium

PGE: Pencil graphite electrode

Poly-(AHNSA): Poly-4-amino-3-hydroxy-1-naphthalenesulfonic acid

Poly-(TERE): Polyterephthalic acid

PVA: Polyvinyl alcohol

SiTi: Amorphous and mesoporous silica/titania

SnO_2_: Tin dioxide

SPCE: Screen-printed carbon electrode

SWV: Square-wave voltammetry

TiO_2_: Titanium dioxide

VG: Vertical graphene

Y-Ni-MOF: Bimetallic yttrium-nickel-based metal–organic framework

ZIF: Zeolitic imidazolate framework

ZnO: Zinc oxide

**Table 4:** Results of simultaneous analysis of EP and NEP in commercial human blood serum (n = 3).

	EP			NEP		
Added EP and NEP (μM)	Calculated EP (μM)	Recovery %	Error %	Calculated NEP (μM)	Recovery %	Error %
50	46.39	94.92	5.08	52.33	97.14	2.86
100	104.36	102.38	2.38	95.86	97.35	2.65
200	196.40	94.74	5.26	198.41	99.30	0.70
